# Progress in AQP Research and New Developments in Therapeutic Approaches to Ischemic and Hemorrhagic Stroke

**DOI:** 10.3390/ijms17071146

**Published:** 2016-07-18

**Authors:** Lauren E. Previch, Linlin Ma, Joshua C. Wright, Sunpreet Singh, Xiaokun Geng, Yuchuan Ding

**Affiliations:** 1 Department of Neurosurgery, Wayne State University School of Medicine, Detroit, MI 48201, USA; lprevich@med.wayne.edu (L.E.P.); jowright@med.wayne.edu (J.C.W.); sunpreet.singh94@gmail.com (S.S.); 2 Department of Neurology, Beijing Luhe Hospital, Capital Medical University, Beijing 101199, China; hotmoonml@hotmail.com; 3 China-America Institute of Neuroscience, Beijing Luhe Hospital, Capital Medical University, Beijing 101199, China

**Keywords:** aquaporin, vasogenic edema, cytotoxic edema, ischemic stroke, hemorrhagic stroke

## Abstract

Cerebral edema often manifests after the development of cerebrovascular disease, particularly in the case of stroke, both ischemic and hemorrhagic. Without clinical intervention, the influx of water into brain tissues leads to increased intracranial pressure, cerebral herniation, and ultimately death. Strategies to manage the development of edema constitute a major unmet therapeutic need. However, despite its major clinical significance, the mechanisms underlying cerebral water transport and edema formation remain elusive. Aquaporins (AQPs) are a class of water channel proteins which have been implicated in the regulation of water homeostasis and cerebral edema formation, and thus represent a promising target for alleviating stroke-induced cerebral edema. This review examines the significance of relevant AQPs in stroke injury and subsequently explores neuroprotective strategies aimed at modulating AQP expression, with a particular focus on AQP4, the most abundant AQP in the central nervous system.

## 1. Introduction

Stroke constitutes a major global health problem, being the second leading cause of death worldwide, and the foremost cause of neurological disability in the United States. There are two types of stroke which are classified based on their etiological mechanism [1]. Ischemic stroke accounts for 80% of stroke cases, and occurs as a result of cerebral vessel occlusion due to a thrombus or embolus. The resulting loss of perfusion disrupts ion homeostasis and impairs cellular metabolism. Conversely, hemorrhagic stroke involves the rupturing of a vessel, causing the leakage of blood into the parenchyma. Cerebral edema development often accompanies both ischemic infarct and intracerebral hemorrhage (ICH), but the mechanism underlying edema formation differs between the two types of stroke. Without medical intervention, brain edema results in increased intracranial pressure, decreased blood flow, and ischemia, and is correlated with poor patient outcomes and a greater incidence of mortality. Currently, the only approved therapies for treating cerebral edema include decompressive craniectomy and osmotherapy, which both aim to alleviate downstream effects, rather than addressing the molecular mechanisms underlying edema development [2].

## 2. Aquaporins

Aquaporins (AQPs) are a class of water channel proteins which have been implicated in the regulation of both physiological and pathological water homeostasis, and thus represent a promising target for alleviating stroke-induced cerebral edema. This highly conserved protein family is present in a variety of different organisms, ranging from bacteria to mammals, with a degree of evolutionary conservation that is likely to be indicative of the importance of these channels. AQPs are embedded within the plasma membrane as tetramers, with each channel being composed of six transmembrane α-helix proteins joined with two half-helices at the N- and C-terminal ends of the protein. This organization forms a central pore, through which water or other molecules pass, depending on the channel subtype. Thus far, 13 mammalian AQPs have been identified to be heterogeneously expressed in various tissues. Seven types of AQPs (AQP1, AQP3, AQP4, AQP5, AQP8, AQP9, AQP11) have been found to be expressed in the mammalian central nervous system (CNS), with AQP4 being the dominant AQP channel present in the mammalian brain [3,4].

### 2.1. Aquaporin 1

AQP1 channels primarily function as mediators of water-transport, but can also pass volatile substances like CO_2_. AQP1 is widely expressed throughout the body, most prevalently in the collecting ducts of the kidney, lungs, red blood cells, and the CNS [5]. In the mammalian brain, AQP1 channels are predominantly located within the apical epithelium of the choroid plexus, and have been implicated in the secretion of cerebrospinal fluid. However, a population of AQP1-expressing astrocytes was recently discovered in the white matter and glia limitans in the brains of non-human primates. This astrocyte population is absent in the rodent brain, indicating that AQP1 expression differs among species [3,6,7] Under pathological conditions, these astrocytic AQP1 channels are thought to have a complementary role to AQP4 in regulating brain water homeostasis. Moreover, in non-human primate brains, AQP1 is also expressed in neurons innervating the pial vasculature, suggesting a potential role in regulating cerebral blood flow [6]. These expressional variations among animal models signify that when developing AQP1-targeted therapies based on preclinical data, potential species-dependent differences in distribution patterns must be considered. Additionally, the diffuse expression patterns of AQP1 throughout the body further complicate drug development, as targeting cerebral AQP1 channels would likely also impact other organ systems.

### 2.2. Aquaporin 9

AQP9 channels are classified as aquaglyceroporins, meaning that in addition to water, the channel also permits the passage of other molecules, including glycerol, urea, lactate, and monocarboxylates [5]. AQP9 channels are primarily found in the liver and testis; however, they are also expressed to a lesser extent in ependymal cells and tanycytes lining the cerebral ventricles, within astrocytes and brainstem catecholaminergic neurons, and in dopaminergic and hypothalamic neurons located in the midbrain [8,9]. Unlike AQP4, which is localized to astrocytic endfeet, AQP9 is found dispersed throughout astrocytic cell bodies and processes. Although the specific role of AQP9 has not yet been elucidated, these channels have been proposed to play a role in regulating brain energy homeostasis, evidenced by its presence on the inner mitochondrial membrane and its expressional sensitivity to plasma insulin levels [10,11]. Akin to AQP1, species-dependent distribution patterns were discovered when localizing AQP9 expression. In primates, populations of AQP9-expressing neurons were observed in additional cortical areas which were absent in rodents [3]. Moreover, like in the case of AQP1, targeting cerebral AQP9 channels would likely come at the detriment of normal physiological functioning in other areas of the body.

### 2.3. Aquaporin 4

AQP4 is a bidirectional water-specific channel which is primarily concentrated within the glial limitans and astrocytic endfeet in association with the vasculature at the division between the brain parenchyma and major fluid compartments, such as the blood brain barrier (BBB). To a much lesser extent, AQP4 has also been found to be expressed in the retina, stomach, kidney collecting duct, and skeletal muscle [5]. AQP4 tetramers are anchored to the plasma membrane via α-syntrophin, an element of the dystrophin protein complex, which contributes to the protein’s polarized astrocytic expression [8]. These tetramers assemble to form large clusters, referred to as orthogonal array particles, which are visible in the outer layer of the BBB. Studies using AQP4-knockout mice established that AQP4 acts as a facilitator for cerebral water transport across pial and ependymal surfaces, as well as through blood vessel walls [5,11]. Additionally, at the BBB, astrocytes utilize AQP4 in order to maintain intracranial fluid homeostasis. Dysregulation of AQP4-regulated water flux has been implicated in a number of neurodegenerative diseases, and AQP4 channels play a particularly complex bimodal role in stroke pathophysiology and edema formation [4]. Gaining a greater understanding of the mechanisms underlying the role of AQP4 in water homeostasis will be beneficial for the development of selective AQP-targeted therapies aimed at mitigating the dysregulation of cerebral water transport and clearance.

### 2.4. Aquaporins 3, 5, 8, and 11

Other AQP subtypes are expressed in brain parenchyma and capillary endothelial cells, but their role in the pathophysiology of stroke and edema remains unclear. Protein and mRNA expression of AQP3, AQP5, and AQP8 were observed in both astrocytes and neurons in a rat model [12]. Further investigation revealed that AQP5 may be involved in the progression of cerebral edema. Two studies have provided evidence that AQP5 expression increased and subsequently decreased following ischemic stroke [12,13].

AQP11 along with AQP12 are members of the superaquaporin subfamily. These AQPs are localized intracellularly and are distantly related to the other AQP protein subtypes. AQP11 was expressed within neurons, choroid plexus epithelium, and brain capillary endothelial cells in a mouse model [14,15]. Further studies are needed to determine whether or not these AQPs contribute to the pathological progression of ischemic or hemorrhagic stroke.

## 3. Cerebral Edema

There are two categories of cerebral edema which are differentiated based on their underlying mechanism and time course: cytotoxic and vasogenic. Cytotoxic edema is cellular swelling which typically manifests within 24–48 h of the initial ischemic or hemorrhagic insult, and involves cellular damage and electrolyte imbalance. The cellular swelling associated with cytotoxic edema is induced following a massive influx of water and osmolites, predominately Na^+^ and Cl^−^, shifting from the interstitial space to intracellular compartments. Following injury, all cell types in the CNS will undergo this process, but astrocytes are particularly affected [2]. Conversely, vasogenic edema is extracellular swelling which typically peaks about 72–96 h after the initial injury, and involves a breakdown of the blood-brain barrier and extravasation of plasma proteins [2,16,17]. While ICH is typically associated with the development of vasogenic edema, in cases of ischemic stroke, edema formation proceeds in a cascade, wherein cytotoxic edema manifests during the first few hours following the ischemic insult. Subsequent prolonged periods of ischemia can prompt the breakdown of the BBB and the hemorrhagic conversion of ischemic tissue, resulting in a progression from cytotoxic to vasogenic edema.

### 3.1. Aquaporins in Vasogenic Edema

**Figure 1:**
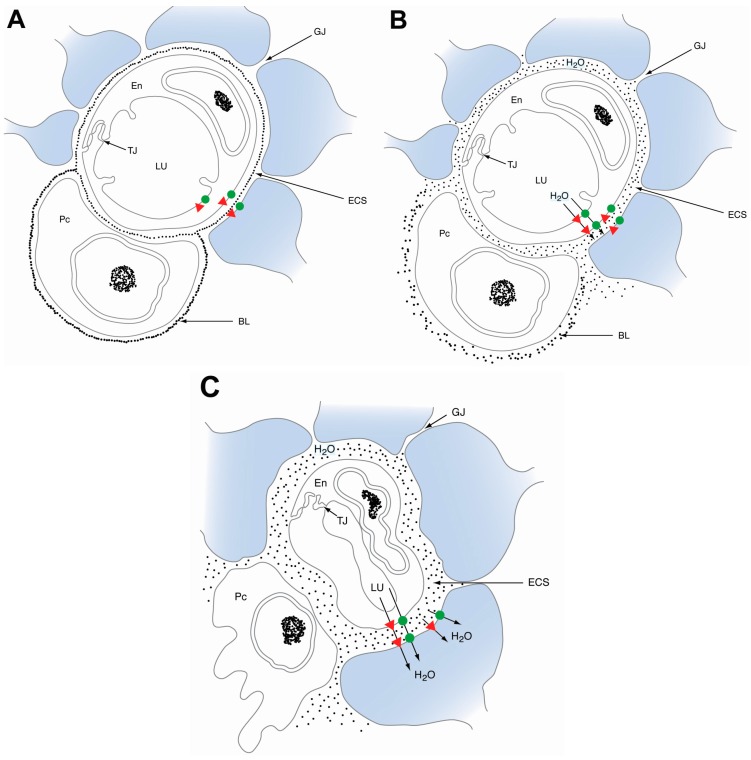
Schematic diagram illustrating the temporal progression of acute edema formation after ischemic stroke. (**A**) Physiologically normal blood brain barrier (BBB); (**B**) in the early stages of ischemic stroke, aquaporin 4 (AQP4; **green dot**) downregulation, and AQP1 (**red triangle**) upregulation, occurs on astrocytic endfeet. Cytotoxic cerebral edema manifests as AQP4 facilitates water passage into the astrocyte compartment (**blue**), causing cellular swelling and disruption of the basal lamina; (**C**) in cases of hemorrhagic stroke and the late stages of ischemic stroke, BBB disruption results in vasogenic edema. Water accumulates in the extracellular space (ECS) after exiting the leaky capillaries. Concurrently, AQP4 channels in the astrocyte endfeet are upregulated, which gradually facilitates the removal of extracellular fluid. BL: basal lamina, En: endothelium, ECS: extracellular space, GJ: gap junction, LU: capillary lumen, Pc: pericyte, TJ: tight junction.

AQP4 plays a role in mediating water influx during the manifestation of edema as well as regulating water efflux during clearance. AQP4 expression is both spatially and temporally regulated based on the type of stroke model, with AQP4 downregulation noted in cytotoxic edema, and an upregulation observed at the onset of vasogenic edema, potentially serving to accelerate water clearance (Figure 1) [2,4,8]. Wang et al. observed that AQP4 was highly expressed around glial cell processes in the perihematomal mouse brain up to 3–7 days post-ICH. In addition, AQP4 deletion in an ICH mouse model exacerbated edema formation and resulted in more severe neurological deficits. These mice exhibited decreased specific gravity of the brain tissue around the area of hematoma, increased brain microvessel damage, and greater terminal deoxynucleotidyl transferase dUTP nick-end labeling (TUNEL) staining, indicative of accelerated neuronal apoptosis [16]. AQP4 temporal expression patterns and the worsening of edema in AQP4-deficient mice are consistent with the concept that vasogenic clearance of water into the cerebrospinal fluid (CSF) and the blood is AQP4 dependent [4]. Altogether, these results suggest that AQP4 upregulation plays a significant role in the formation of vasogenic edema following both hemorrhagic and late ischemic stroke. As in the case of AQP4, AQP1 and AQP9 are both upregulated following ICH [18,19]. However, the functional consequences of this increased expression during vasogenic edema have not yet been explored.

### 3.2. Aquaporins in Cytotoxic Edema

Conversely, AQP4 plays an opposing role in cytotoxic edema. AQP4 expression was found to be decreased at 24 h after ischemic insult in focal cerebral ischemic mouse models, with a partial recovery observed around 72 h [20,21]. This downregulation may be a protective, but ineffectual, response in order to counteract the influx of water and resultant cerebral swelling. Furthermore, unlike in models of vasogenic edema, AQP4 knockout mice exhibited reduced infarct sizes, decreased brain water content, and improved survival and neurological outcomes. The neuroprotection conferred from AQP4 deficiency in models of cytotoxic edema is likely due in part to a reduced rate of water infiltration into the parenchyma [20,21].

AQP9 is significantly upregulated in astrocytes around the periphery of cerebral infarcts 24 h after transient middle cerebral artery occlusion (MCAO) in mice, and levels continue to increase until peaking at seven days post-stroke [22]. However, unlike AQP4, AQP9 induction was not found to be correlated with swelling of the parenchyma, suggesting that it may not play a significant role in the mediation of water flux following ischemic insult [18,20]. Instead, this expressional time course and its facilitation of lactate transport between astrocytes and neurons suggests that AQP9 may play a larger role in energy metabolism and glial scar formation following ischemic injury [4,5,18].

AQP1 expression is also reported to be upregulated following ischemic insult. Kim et al. [23] found that AQP1 is highly expressed in cortex and striatal endothelial cells 22 h post-MCAO. However, it remains unclear if the increased expression of AQP1 is correlated with the progression of cytotoxic edema development. The limited expression of AQP1 within the CNS suggests that although these channels may work concomitantly with AQP4 channels to contribute to the dysregulation of water homeostasis, AQP4 is the principal regulator of edema formation [3]. Moreover, hypertonicity has been demonstrated to affect AQP4, but not AQP1 expression, suggesting that AQP1 and AQP4 are subject to different mechanisms of regulation [24].

## 4. Aquaporin Drug Therapies

Due to their integral role in brain edema formation and resolution, AQPs are promising therapeutic targets to mitigate the damage of ischemic and hemorrhagic stroke. AQP4 is especially attractive because of its prime location for water exchange, between brain parenchyma and the circulatory system. Many AQP4 modulators have been discovered in recent years using a variety of animal and cellular models (Table 1). The current challenge is translating these therapies into clinical applications.

Clinical utilization of AQP4 modulators is complicated by the bimodal role that AQP4 plays in stroke progression. The onset of vasogenic edema, which develops during hemorrhagic stroke and late stages of ischemic stroke, is associated with the upregulation of AQP4 channels. Conversely, cytotoxic edema, which appears during the early stages of ischemic stroke, is correlated with a downregulation of AQP4 channels. Therefore, AQP4 inhibitors may be beneficial during the early stages of stroke, but could be deleterious if administered during the later stages when the clearance of water from the brain into the vasculature is crucial [68]. This review summarizes the therapies that modulate AQP4 expression or permeability.

### 4.1. Hormones

Several recent studies have shown that endogenous hormone levels can modulate AQP4 expression. Animal models of stroke and brain edema have indicated arginine vasopressin V1 (AVPV1), erythropoietin, estrogen, progesterone, melatonin, and thyroid hormone as possible targets.

#### 4.1.1. Thyroid Hormone

Thyroid hormone (T3; 3,3′,5-triiodo-l-thyronine and T2; 3,5-diiodo-l-thyronine) has also been observed to confer neuroprotective effects via AQP4 modulation [33,69]. In an MCAO mouse model, T3 and T2 administered post-stroke mitigated edematous swelling via the downregulation of AQP4 expression. Additionally, the treatment groups demonstrated a significant improvement in neurological functioning [33]. The dosage administered was sufficient to transiently elevate levels of thyroid hormone, but insufficient to induce thyrotoxicity or hyperthyroidism in mice. In addition to encouraging preclinical results, there is considerable human clinical evidence to indicate that low T3 is a predictor of worse outcomes following stroke, suggesting that exogenous supplementation of thyroid hormone may be a promising neuroprotective therapy for the treatment of ischemic stroke [33,69].

#### 4.1.2. Melatonin

Melatonin is a tryptophan metabolite secreted by the pineal gland that has been observed to confer neuroprotective effects through the inhibition of AQP4 in models of early cerebral ischemia [22,70,71]. Melatonin functions as an antioxidant and free radical scavenger, and its small size and high liphophilicity allow for crossing of the BBB, a characteristic which is highly beneficial in the development of neurotherapeutics. Melatonin administration limited astrocytic swelling and enhanced glial cell survival in MCAO rat models [70,71]. Furthermore, melatonin administration at the start of reperfusion 1 h post-MCAO significantly limited edema formation, and resulted in reduced neuronal apoptosis, decreased infarct volumes, as well as improved motor coordination and neurological functioning. Cortex and striatal AQP4 mRNA and protein expression levels were significantly decreased at 24 h in the melatonin treatment group when compared to ischemic controls. Additionally, melatonin administration enhanced levels of protein kinase C (PKC), a proposed phosphorylation site of AQP4, which is thought to modulate its water permeability and hinder AQP4 upregulation in response to ischemic conditions. Therefore, melatonin may alleviate cytotoxic cerebral edema by acting as an activator of PKC, and thus by promoting AQP4 inhibition indirectly [22].

### 4.2. Loop Diuretics

In addition to their primary effect, new studies indicate that loop diuretics significantly modulate expression of aquaporin 4. One of the most promising drugs is bumetanide, a sulfamoyl loop diuretic commonly prescribed in cases of cardiac and renal failure, which has been increasingly used in the experimental treatment of various neurological disorders [72]. Intravenous bumetanide administration has been observed to block AQP4 channels and edema formation, as well as to reduce infarct volumes in an adult rat MCAO model. However, bumetanide is also an inhibitor of Na^+^–K^+^–2Cl^−^ cotransport (NKCC), the impediment of which has also been implicated in the attenuation of ischemia-induced cerebral edema [7,34]. Therefore, the efficacy of bumetanide could potentially be attributable in part to vascular endothelial cell NKCC inhibition. Despite promising preclinical data, clinical trials employing bumetanide in an effort to ameliorate neurological symptoms have encountered concerning problems, including poor BBB penetration and adverse systemic side effects, such as diuresis, hypokalemic alkalosis, and hearing loss [72].

### 4.3. Miscellaneous Drugs

Piroxicam is a non-steroidal anti-inflammatory drug (NSAID), commonly prescribed for rheumatoid arthritis and musculoskeletal disorders, that has been reported to inhibit AQP4 expression and resultant edema formation in models of cerebral ischemia-reperfusion injury [48,73,74]. When piroxicam was administered 30 min prior to the induction of focal cerebral ischemia in an MCAO rat model, it resulted in significant reductions in brain water content and infarct volumes, decreased neurological deficit scores, and improved motor functioning when compared to ischemic controls. In addition, piroxicam-treated groups displayed decreased AQP4 protein levels in the cortex and striatum, areas which are particularly sensitive to ischemic injury, when compared to controls [48].

Propofol, a widely used intravenous anesthetic, has been reported to attenuate AQP4 expression [75,76]. When administered shortly after traumatic brain injury (TBI) induction, propofol reduced the expression of known AQP4 activators, IL-1β and TNF-α, and as a result, downregulated AQP4 expression [76]. Additionally, edavarone, a free radical scavenger, reduced AQP4 protein levels and immunoreactivity around the area of infarction when administered 24 h after reperfusion in an MCAO rat model [41]. Edavarone has also been reported to significantly reduce cortical edema, infarct volume and neurological deficit scores in focal ischemic stroke animal models [40,41,77].

### 4.4. Preconditioning

Exposing the brain to sub-toxic levels of ischemia or chemicals modulating oxidative cell respiration may precondition cells for future ischemic events. Many recent studies have shown that the effects of preconditioning are often mediated by AQP4 expression. Preconditioning constitutes a relevant neuroprotective approach for patients who are undergoing invasive or lengthy surgeries, like a coronary artery bypass graft or a carotid endarterectomy, in which there is the potential for ischemic exposure [78]. Pre-conditioning strategies could be beneficial across the population for decreasing stroke mortality risk, and in surgical patients to improve post-surgical outcomes.

#### 4.4.1. Chemical Preconditioning

Chemical preconditioning has been explored in recent years with studies using thrombin and 3-nitroproprionic acid. Low-doses of these substances delivered over weeks prior to MCAO greatly attenuated the subsequent ischemic event and reduced levels of AQP4 [55,57]. The authors hypothesized that these stressful conditions activate endogenous protective mechanisms for ischemic events, which may be conserved across mammalian species.

#### 4.4.2. Hyperbaric Oxygen Preconditioning

Hyperbaric oxygen preconditioning (HBO-PC) has also been proposed as a means of alleviating cerebral edema via AQP4 inhibition. HBO-PC has been observed to decrease brain water content and AQP4 expression in stroke models [61,79,80]. Moreover, treatment with HBO-PC in a focal cerebral ischemic rat model resulted in decreased infarct volumes and improved sensorimotor function when compared to ischemic controls [79]. HBO-PC also appears to play a neuroprotective role in the progression of hemorrhagic stroke, resulting in the downregulation of AQP4 expression around the hemorrhagic focus and the prevention of edema formation in an ICH rat model. However, the HBO-PC treatment group exhibited no significant reduction in neurological dysfunction when compared to ICH controls, indicating that HBO-PC may be more suitable in the treatment of ischemic stroke due to its limited therapeutic potential in alleviating hemorrhagic stroke symptomology [61]. 

#### 4.4.3. Remote Limb Ischemic Preconditioning

Remote ischemic post-conditioning (RIPC) constitutes an alternative noninvasive neuroprotective approach which has been purported to suppress AQP4 expression, and thus limit edema formation in models of focal cerebral ischemia [62,81,82]. RIPC proposes that recurrent, rapid exposures to brief ischemia in the early phase of reperfusion can induce tolerance to a more severe long-term ischemic insult, in the same or even a remote organ. Employing hindlimb clamping to occlude blood flow post-MCAO resulted in a decreased number of AQP4-positive cells and reduced AQP4 mRNA levels at 24 h in comparison to MCAO controls [81,82]. Additionally, RIPC suppressed activation of the transcription factor nuclear factor-κB (NF-κB), a known activator of AQP4, suggesting that the therapy may modulate AQP4 expression by downregulating the NF-κB pathway [82]. RIPC also limited parenchyma water content, attenuated the swelling of astrocytic foot process, decreased infarct volumes, and precluded BBB disruption [62,82]. Although RIPC has shown promise in experimental models of cerebral ischemia, it failed to have any influence on AQP4 expression levels and did not confer any significant beneficial effects in an ICH rat model at either 24 or 72 h post-surgery [83].

### 4.5. Neurotransmitters

Agmatine is an endogenous metabolite of L-arginine which has been shown to confer neuroprotective effects in models of ischemia via the inhibition of AQP1, AQP4, and AQP9. Agmatine administration immediately following ischemic insult limited BBB disruption and reduced brain water content at 24 h post-injury in an MCAO mouse model [23,35]. Although agmatine has yielded promising results in experimental ischemic models thus far, as previously discussed, the diffuse distribution patterns of AQP1 and AQP9 make them less attractive targets for targeted therapy development due to the potential for unwanted side effects. The efficacy of agmatine in attenuating ICH-induced vasogenic edema has not yet been evaluated.

### 4.6. Metabolism-Attenuating Therapies

#### 4.6.1. Ethanol

Another approach to mitigating the damage of ischemic stroke is to induce a hibernation-like state of low metabolic activity. Recent studies revealed that ethanol decreases cerebral edema and improves BBB integrity following ischemic stroke [42,43]. When low-dose ethanol was administered to rats following an MCAO, a significant decrease in AQP4 expression was observed compared to the non-treatment stroke group. The mechanism by which ethanol produces these beneficial changes is yet to be clarified. However, it was hypothesized that the effect was related to a general decrease in metabolic activity across the brain [42]. Minimizing cellular metabolism may be responsible for mitigating AQP4 expression.

#### 4.6.2. Therapeutic Hypothermia

There is considerable evidence in animal models to indicate the feasibility of using mild to moderate therapeutic hypothermia (TH) (30–34 °C) in the treatment of stroke [84,85,86,87]. However, the mechanism underlying the efficacy of TH is poorly understood. One proposed area of action is the mitigation of order to alleviate vasogenic cerebral edema in an ICH rat model. In order to simulate vasogenic edema resulting from ICH, thrombin injections were administered immediately following surgery. TH was subsequently induced in the treatment group directly after injections. The TH group displayed a reduction in basal ganglia water content and significantly decreased BBB extravasation when compared with normothermic ICH controls (37 °C). Notably, TH did not alter the BBB permeability in normal tissue. Furthermore, in comparison to the normothermic group, the TH group was found to exhibit an increase in AQP4 mRNA and protein levels beginning at 24 h that continued to increase until peaking at 48 h [88]. These results suggest that focal mild TH may be neuroprotective in cases of ICH, in part, via the upregulation of AQP4, and thus leading to more effective clearance of vasogenic edema.

The efficacy of mild TH was also evaluated in a porcine model of cardiac arrest and resuscitation, which is known to generate whole body ischemia-reperfusion injury, and can result in the formation of cytotoxic cerebral edema. After being subjected to eight min of untreated ventricular fibrillation (VF), followed by the induction of TH with a cooling device, the treatment group exhibited markedly higher survival rates at 24 and 72 h following ischemic insult when compared to normothermic controls. Swine neurologic deficit scores were also significantly lower at 24 and 72 h in the TH group than in the normothermic group, indicative of improved neurological functioning in the treatment group. A decrease in intracranial pressure, an indicator of cerebral edema, and an increase in cerebral blood flow were also observed in the TH group. In the cerebral cortex, AQP4 protein levels were decreased at 24 h in the treatment group when compared to controls. Furthermore, the AQP4-activator NF-κB was downregulated at 24 h in the TH group. However, there was no difference detected between the two groups at 72 h. These results indicate that TH may also be beneficial in the treatment of ischemic stroke via the downregulation of AQP4, resulting in diminished formation of cytotoxic cerebral edema [59].

Moreover, transarterial regional hypothermia was found to be neuroprotective in a rat MCAO model. Regional hypothermia was induced with a cold saline infusion prior to reperfusion. The TH group exhibited reduced infarct volumes and BBB disruption in comparison to controls. In the MCAO control group, AQP4 was upregulated at 2 h, preempting astrocytic endfoot swelling at 6 h. This swelling impinged on adjacent microvessels, accelerating infarct progression, and further propagating ischemic injury. However, AQP4 expression was decreased, astrocyte swelling was limited, and these microvasculature changes were almost absent in the cold saline group. The upregulation of AQP4 was the earliest detectable event in the time course analysis, signifying that its inhibition may be crucial for precluding microvasculature disruption and the subsequent activation of inflammatory mediators in the ischemia-reperfusion cascade [58]. This provides further evidence that TH represents one of the most promising treatment strategies in conferring neuroprotective effects following stroke.

## 5. Future Directions

As discussed above, the therapies for treating cerebral edema currently aim to alleviate downstream effects, rather than addressing the molecular mechanisms underlying edema development. The development of suitable therapeutic interventions poses a complex problem due to the bimodal role of AQP4 in edema progression and its opposing actions in ischemic and hemorrhagic stroke. Although many of the agents presented here have seen success in experimental models, further research is necessary to assess their efficacy and to identify any unwanted side effects which may manifest as a result of therapy administration. Guidelines for the timing of drug delivery must also be established before clinical application is feasible. Nevertheless, promising results from preclinical studies warrant additional efforts. Furthermore, our understanding of the role AQP1 and AQP9 vasogenic edema development and clearance is particularly limited, and this represents an additional avenue for future investigation.

## Figures and Tables

**Table 1:** Summary of evidence for potential stroke therapies that alter aquaporin 4 (AQP4) viability in astrocytes.

Therapy	Aquaporin-4 Expression (after Stroke and Treatment)	Model	Time Points (Reperfusion Time)	References	Year
**Drug Therapies**					
Arylsulfonamides					
4-acetamido-benzsulfonamide	Decreased permeability	*Xenopus*oocytes	N/A	Huber et al. [25]	2007
Acetazolamide (AZA)	Decreased permeability	*Xenopus*oocytes	N/A	Huber et al. [25]	2007
	Decreased permeability	Aquaporin 4 proteins within liposomes	N/A	Tanimura et al. [26]	2009
6-Ethoxy-benzothiazole-2-sulfonamide (EZA)	Decreased permeability	*Xenopus*oocytes	N/A	Huber et al. [25]	2007
Hormones and hormone receptor modulators					
Arginine vasopressin (AVP) V1 receptor antagonist (SR 49059)	Decreased	Mouse transient middle cerebral artery occlusion (MCAO), 60 min	24 h	Liu et al. [27]	2010
	Decreased	Rat transient MCAO, 2 h	2 h	Okuno et al. [28]	2008
Erythropoietin	Decreased	Mouse primary brain edema	N/A	Gunnarson et al. [29]	2009
Estrogen	Decreased	Mouse transient MCAO, 30 min	3 days	Shin et al. [30]	2011
Melatonin	Decreased	Rat transient MCAO, 1 h	24 h	Bhattacharya et al. [22]	2014
Neuregulin-1β	Decreased	Rat transient MCAO, 90 min	0.5, 1, 1.5, and 2 h	Li et al. [31]	2008
Progesterone	Decreased	Rat astroglial cell culture, 3 h hypoxia	N/A	Habib et al. [32]	2014
Triiodothyronine	Decreased	Mouse transient MCAO, 60 min	24 h	Sadana et al. [33]	2015
Na^+^/K^+^/Cl^−^ channel blockers (loop diuretics)					
AqB013 (4-aminopyridine carboxamide analog)	Decreased aquaporin-4 permeability	Xenopus laevis oocyte	N/A	Migliati et al. [7]	2009
Bumetanide	Decreased permeability	Xenopus oocytes	N/A	Migliati et al. [7]	2009
	Decreased	Rat transient MCAO, 90 min	24 h, and 2 days	Migliati et al. [34]	2010
Furosemide	Decreased aquaporin-4 permeability	Xenopus laevis oocyte	N/A	Migliati et al. [7]	2009
Neurotransmitters and neurotransmitter modulators					
Agmatine	Decreased aquaporin-4 positive cells	Rat transient MCAO, 90 min	4 days	Wang et al. [35]	2010
Ifenprodil	Decreased	Rat cardiac arrest model	N/A	Xiao et al. [36]	2005
Other organic molecules					
Astragaloside IV	Decreased	Rat transient MCAO, 90 min	24 h	Li et al. [37]	2013
Carvacrol	Decreased mRNA and protein levels (perihematomal area)	Rat intracerebral hemorrhage	3 days	Zhong et al. [38]	2013
Dexamethasone	Decreased mRNA levels (perihematomal area)	Rat intracerebral hemorrhage	24 h	Gu et al. [39]	2007
Edaravone	Decreased	Rat transient MCAO, 90 min	24 h	Kikuchi et al. [40,41]	2009
Ethanol	Decreased	Rat transient MCAO, 2 h	3 and 24 h	Zeng et al. [42,43]	2012
Ginsenoside Rg1	Decreased	Rat transient MCAO, 2 h	6, 24 h, 3, 7, and 14 days	Zhou et al. [44,45]	2014
Hydrogen sulfide	Decreased	Rat transient MCAO, 2 h	24 h	Wei et al. [46]	2015
Phorbol myristate acetate (PMA)	Decreased	Rat transient MCAO, 2 h	2 h	Okuno et al. [28,47]	2008
Piroxicam	Decreased	Rat transient MCAO, 1 h	24 h	Bhattacharya et al. [48]	2013
Probenecid	Decreased	Mouse transient MCAO, 1 h	48 h	Xiong et al. [49]	2014
Propofol	Decreased	Rat transient MCAO, 2 h	24 h	Ji et al. [50]	2015
	Decreased	Rat transient MCAO, 1 h	24 h	Lee et al. [51]	2013
Simvastatin	Decreased	Rat transient MCAO, 1 h	24 h	Zhu et al. [52]	2014
TGN-020	Decreased	Mouse transient MCAO, 2 h	24 h	Igarashi et al. [53,54]	2011
Pharmacological preconditioning					
3-Nitroproprionic acid	Decreased	Rat transient MCAO, 2 h	4 days	Hoshi et al. [55,56]	2011
Thrombin	Increased	Mouse transient MCAO, 30 min	1, 24, and 2 day	Hirt et al. [57]	2009
**Non-Drug Therapies**					
Therapeutic hypothermia					
Internal carotid endovascular infusion	Decreased	Rat transient MCAO, 2 h	0, 2, 6, 24 h	Kurisu et al. [58]	2015
External jugular endovascular infusion	Decreased	Pig cardiopulmonary resuscitation	24 h was significant	Zhao et al. [59]	2012
Stress preconditioning					
Exercise pre-training	Decreased	Rat transient MCAO, 90 min	1, 2.5, 7.5 h, 1, 2, 3 days	He et al. [60]	2014
Hyperbaric oxygen preconditioning	Decreased	Rat intracerebral hemorrhage	24 h, 2, 3, 5, and 7 days	Fang et al. [61]	2015
Remote limb ischemic preconditioning	Decreased	Rat transient MCAO, 2 h	24 h	Han et al. [62]	2015
**Other Therapies**					
GV20 and ST36 acupuncture	Decreased	Rat transient MCAO, 2 h	24 h	Xu et al. [63]	2014
Mesenchymal stem cells	Decreased	Mouse transient MCAO, 90 min	24 h, and 3 days	Tang et al. [64]	2014
RNA targets					
miRNA-130a	Decreased	Rat transient MCAO, 60 min	24 h	Sepramaniam et al. [65]	2012
	Decreased	Human astrocytoma cell culture	N/A		
miRNA-29b	Increased	Mouse transient MCAO, 3 h	24 h, and 3 days	Wang et al. [66]	2015
miRNA-320a	Decreased	Rat transient MCAO, 60 min	24 h	Sepramaniam et al. [67]	2010
	Decreased	Human astrocytoma cell culture	N/A		

## Abbreviations

AQP	aquaporin
BBB	blood brain barrier
ICH	intracerebral hemorrhage
MCAO	middle cerebral artery occlusion
HBO-PC	hyperbaric oxygen preconditioning
TH	therapeutic hypothermia
RIPC	remote limb ischemic preconditioning
